# Augmented video consultations in care homes during the COVID-19 pandemic: a qualitative study

**DOI:** 10.3399/BJGPO.2022.0073

**Published:** 2022-09-21

**Authors:** Lorraine Ochieng, Mahan Salehi, Rebecca Ochieng, Dewy Nijhof, Richard Wong, Vinay Gupta, Rishabh Prasad, Bertha Ochieng

**Affiliations:** 1 Sheffield Teaching Hospitals NHS, Sheffield, UK; 2 De Montfort University Faculty of Health & Life Sciences De Montfort University The Gateway, Leicester, UK; 3 University Hospitals of Leicester NHS Trust, Leicester, UK; 4 Willows Health Willows Health, Leicester, UK

**Keywords:** video consultation, COVID-19, remote consultation, care homes, older adults, virtual ward round, primary health care

## Abstract

**Background:**

The COVID-19 pandemic necessitated an unprecedented implementation of remote consultations in UK primary care services. Specifically, older adults in care homes had a high need for infection prevention owing to their existing health conditions. GP practices in the East Midlands incorporated augmented video consultations (AVC) with the potential to support remote healthcare assessments for older adults at care homes.

**Aim:**

To explore GPs' and care home staff's experiences of the use of AVC as a mechanism to perform remote examinations of older adults in care homes.

**Design & setting:**

Qualitative interviews were conducted with GPs and care home staff in the East Midlands, UK, during May–August 2020.

**Method:**

A convenience sample of GPs (*n* = 5), nurses (*n* = 12), and senior healthcare assistants (*n* = 3) were recruited using a purposive approach. Data were collected through semi-structured telephone interviews and analysed using framework analysis.

**Results:**

Findings from participants indicated that AVC enabled real-time patient examinations to aid diagnosis and promoted person-centred care in meeting the needs of older adults. The participants also discussed the challenges of video consultations for patients with cognitive impairment and those receiving end-of-life care.

**Conclusion:**

AVCs show great potential in terms of GPs providing primary care services for care homes. However, healthcare staff must be involved in the development of the technology, and consideration should be given to the needs of older adults with cognitive impairment and those receiving end-of-life care. It is also vital that training is available to encourage confidence and competency in implementing the technology.

## How this fits in

GP practices are experiencing challenges in accessing and providing care to an increasingly ageing population with complex medical needs, necessitating seeking new approaches to handle primary care demand in a flexible, efficient, and cost-effective way. The use of AVC could aid GPs to provide person-centred care to older adults who need medical attention in care homes, reducing the stress of the older adults travelling to GP surgeries. A robust infrastructure, with reliable access to software and internet-appropriate training for clinicians and the multidisciplinary team, is likely to facilitate the uptake and use of AVC in primary care, and meet the needs of an increasingly ageing population.

## Introduction

The ongoing COVID-19 pandemic necessitated the requirement for clinically vulnerable individuals to adhere to stricter measures than those advised to the general public. The *Stay-at-Home, Protect the NHS, Save Lives* campaign,^1^ followed by guidance from the British Geriatric Society,^2^ led care homes to consider the feasibility of managing residents entirely within their rooms throughout the COVID-19 pandemic. For care homes, the challenges from COVID-19 increased multifold, not only owing to the possible risk of transmission of such a virus to an already frail older population, but also owing to the difficulties for the staff that look after patients or residents, including the GPs managing patients in the care homes. In this population, infections may easily be mistaken for worsening existing conditions. Moreover, older adults may present with atypical infection presentations, even in the absence of a fever.^3^ In addition to the increased risk of infection and severe disease progression, many older adults in care homes require support or assistance with their personal hygiene to stop infection transmission.^4^ Studies have shown a particular vulnerability to respiratory illness outbreaks in long-term care settings.^4,5^ As such, care home residents face a greater risk of being infected with COVID-19.

Even before the pandemic, GP practices had been experiencing challenges in accessing and providing care to an increasingly ageing population with complex medical needs. Well-recognised demographic changes describe an ageing population associated with increasing frailty and people with multiple long-term conditions. Indeed, older adults living with combinations of frailty and multimorbidity have some of the highest levels of health and social care needs in the population.^6–8^ Older adults and those described as clinically vulnerable were required to shield against SARS-CoV-2 infection.^9,10^ The combination of complex health needs and increased susceptibility to infection among older adults necessitated the need for faster GP access, irrespective of the resultant social distancing measures that were put in place owing to COVID-19.

The *NHS Long Term Plan* ^11^ sets out ambitions for improvement over the next decade, including underlining the importance of technology in the future of the NHS and establishing the critical priorities that will support digital transformation and provide a step change in the way the NHS cares for citizens. Remote physical examinations through video consultations can help ensure continuity of care while decreasing the risk of SARS-CoV-2 exposure. Studies on the views of those involved with remote digital technology consultations demonstrate a general positivity towards the technology.^12–15^ A qualitative interview study revealed that when remote digital consultations were used for patients with existing long-term conditions, GPs felt confident going forward because of their knowledge of the patient.^16^ Especially during the COVID-19 pandemic, this technology may be of particular use as it allows GPs to manage patients with a high need for primary care services while maintaining measures set in place for care homes. Several studies reported a positive reception of video consultations^17–20^ although few have focused on remote physical consultations by GPs providing care to older adults in care homes.

Virtual reality (VR) and augmented reality (AR) consultations can enhance the real-time consultation process between the doctor and patient at different sites. A study using AR whereby patients were assessed by two independent clinical toxicologists — with one doctor on site performing the physical examination, while the other was remote but able to view and hear the physical examination being done — found high inter-rater agreement; however, the results also identified areas where agreement was limited (possibly owing to streaming quality).^21^ Another study that examined the use of AVC in performing remote physical examinations identified several challenges that arose during the consultations, which could affect both the effectiveness of the physical examination during the video consultation and the patient outcomes.^22^ However, opportunities to examine AVCs are limited, especially in primary care, such as care homes.

Given the challenges of COVID-19, and an increasingly ageing population requiring care, several GP practices incorporated remote video assessment technology into their services, which had the potential to support GPs’ consultations with residents or patients at care homes during and after the COVID-19 pandemic. Remotely accessing basic observations and data about the patient would be vital in the overall assessment and management of the patient. This study sought to explore the experiences of GPs and care home staff when interacting with AVC technology to facilitate the remote physical examination of older adults in care homes. The AVC technology used by GPs and care home staff was called the Medicspot.^23^ This technology allows care home staff to access GPs from a different location. The consultation is performed as a video chat between the GP, the care home staff, and the older adult; however, by using remote diagnostic technology, the GP can also engage in a virtual physical examination with support from care home staff by capturing real-time vital signs and using a stethoscope or otoscope to enhance the consultation further.

The Medicspot station used in this study allowed alignment with the British Geriatric Society’s^2^ recommendations for managing COVID-19 in care homes, which included the following: (i) measurement of vital signs such as blood pressure, heart rate, pulse oximetry, and respiratory rate, where possible; and (ii) checking the temperature when suspecting a patient of COVID-19, thus enabling external healthcare practitioners to triage and prioritise support of residents according to their needs. This article presents findings from a larger study exploring the use of AVC as a mechanism to perform remote examinations with older adults in care homes during the COVID-19 pandemic. The focus is on the experiences of the care home staff and the GPs who used the AVC.

## Method

### Design and context

This article presents the experiences of GPs and care home staff who used AVC as a mechanism to perform remote examinations with older adults in care homes during the COVID-19 pandemic.

### Recruitment of participants

The participants comprised senior healthcare assistants (*n* = 3) and care home nurses (*n* = 12) located within six care homes, and five GPs. The principles of purposive sampling were applied to identify the participants,^24^ recruiting them from care homes and GP practices that had implemented the AVC in Leicester city, in the UK. As part of the inclusion criteria, participants had to be working at one of the participating care homes or GP practices, and using the Medicspot AVC. All participating care homes (*n* = 6) and GP practices (*n* = 9) were sent an information sheet via email and asked to contact the lead researchers in order to participate.

### Data collection methods

Following the taking of consent, qualitative data were collected through semi-structured interviews aimed at evaluating the experience of care home staff and GPs using remote monitoring during the COVID-19 pandemic in Leicester. A literature review highlighted the need to examine a number of factors that would inform the experiences of GPs and nurses working in care homes using the AVC. The development of the tool was also discussed with the research team and colleagues who are experts in digital health. The concepts derived from both the discussion and literature review formed a basis for the topics on the interview schedule, including:

- Experience of using the technology to remotely examine older adults in care homes.
- The advantages and disadvantages of the technology, including any challenges of using the device.
- Effectiveness of the AVC in assisting with diagnosis and management of ill health among older adults in care homes.

Owing to the nature of the lockdown and social distancing requirements, consent and data collection was undertaken through telephone interviews. The one-to-one interviews took place in May–June 2020 and were conducted by the first and second author at a time suiting the participants, with each interview lasting 45–60 minutes. The interviews were audio-recorded and carried out as a form of discourse, in which questions were asked by the interviewers to broaden and elucidate the content in order to achieve a broader perspective of the participants’ experiences. Transcription was verbatim, to ensure that the representation of the interview process and content aligned with the participants’ views.

### Data analysis

A framework approach was used to analyse the data. This approach is a grounded and generative analytical procedure that uses distinct connected stages and involves detailed familiarisation with the data, identification of key themes, and interpretation of the findings within the context of other research, as well as policy and practice considerations.^25,26^ A number of themes had initially been developed by the research team in the course of the ongoing theoretical reflections during fieldwork; these were used for initial coding of the interview materials. This ensured a systematic and rigorous approach to analysis.^25^ The emerging findings were then presented at two local practitioners' online forums organised to discuss the role of digital heath during lockdown, which also included participants. The approach allowed participants and other practitioners to voice their opinions, and reinforced the credibility of the findings. There were no significant differences between the three groups of staff (GPs, nurses, and senior healthcare assistants) regarding their experiences of AVC. Therefore, in presenting the findings, where differences have emerged, these have been highlighted.

## Results

The analysis resulted in the identification of four main themes:

- Remote patient–doctor communication during a pandemic.
- Real-time patient examination in care homes during lockdown.
- Person-centred care in meeting the needs of older adults in care homes.
- Optimising remote consultation and physical examination of older adults in care homes.

### Remote patient—doctor communication during a pandemic

A key advantage reported by all participants — that is, by GPs, nurses, and senior healthcare assistants (identifiers used in quotes are Dr, Nurse, and Carer) — is that the AVC allowed a rapid GP consultation and timely treatment for older adults. They reported that the use of AVC was a good way of maintaining contact with GPs while observing social distancing measures during the COVID-19 pandemic. Nurses and senior healthcare assistants indicated that the use of AVC eliminated the waiting time for a response from GPs to remotely examine the patient:

*' … Before Medicspot, we would have to wait even if there was need for an urgent GP visit. We would have to wait for 6 to 8 hours for a visit using out-of-hours services you could even be looking at 14 hours for a visit specially at the moment. With the Medicspot, the GPs are there and every time we have used it, so there is no delay in treatment or examination.'* (CH_Nurse_01)

GPs indicated that they were able to remotely conduct real-time physical examination, which also enabled them to physically see the patient and their reactions to the examination:

*'Anytime I use Medicspot is a time when I would have had to go see the patient or send one of my colleagues to go and see the patient.'* (CH_DR_01)

*'It is nice to see your patient and for them to see me. To get that, you know, visual feedback, you see their facial expressions and appreciation, or see them register their concern on their face or the pain on their face, which helps when prescribing medication for them and monitoring their condition.'* (CH_DR_03)

In addition, it allowed multiple virtual GP visits during a pandemic:

*'In some homes, we’ve set up what we call proactive virtual ward rounds. So, we try and give some, er, time that the staff can routinely book people in who they’re worried about. They can still phone up about emergencies on the day, but they can book in people that start to get little niggles or worried about that they might get out of control.* […] *We’ve advised that the virtual wards are for people who have … start exhibiting reduced eating and drinking, reduced physical activity, these so-called soft signs. So, we’ve tried to give them a prompt.'* (CH_DR_04)

### Real-time patient examination in care homes during lockdown

The AVC enabled engagement with older adults in the care homes. Feedback from GPs identified that the AVC assisted the diagnosis process by taking real-time vital signs and measurements. In addition, it allowed GPs and care home staff to ensure that, where appropriate, the older adult was involved in the consultation and understood the advice given:

*'I was able to hear her chest remotely, by me using the device so erm, that was good. It’s good that, erm, I was able to see her appearance from the camera as well, I was also able to have direct discussion with the patient as well and not through the staff only. The device gives one a greater level of depth and detail to the examination process.'* (CH_DR_03)

The GPs and care home staff stated that the tools provided with the specific AVC were useful for expediting the treatment process. As the GP was able to conduct a remote examination, decisions were implemented in a timely manner and prescriptions were received a lot faster than before. The GPs indicated the significance of visualising their patient and their body language during the consultation:

*'So, I normally use the device for a number of reasons. The main reason is to use the high definition camera to* [look] *into the ear, nose and throat or look at skin lesions. Or I use it for the auscultation tool, so I can listen to the chest when I’m suspecting any chest infection or any problems with the heart itself* […] *if you get a Bluetooth device that has a stethoscope that connects to my iPhone I could do a FaceTime call or a WhatsApp call and I could listen remotely to heart sounds and the chest. If you develop that, which will probably be a lot cheaper than your big Medicspot device, patients could potentially even buy that for themselves, have it at home and* [the] *doctor could listen to their chest, nurse could listen to their chest if they needed* [to]*.'* (CH_DR_01)

The GPs and care home staff also identified other advantages benefiting the older adult residents. The participants believed that the device gave the older adult patient in a care home the opportunity to see and speak to the GP themselves, unlike with the telephone consultations, where the care home staff are the only ones who speak to the GPs. Interestingly, the care home staff described how AVC increased confidence in treatment because the older adult patient was able to interact with the GP virtually, which helped to create a better personalised treatment plan:

*'I feel it’s more personal. So as I said, pre-Medicspot we would speak to a GP over the telephone and it would generally be a member of staff. The residents wouldn’t necessarily get to talk directly to them. Whereas this way, not only does the GP get to see somebody, especially during the times that they can’t visit, they get to see the residents, see how they are presenting. The residents get to see them, which means they know whom they are talking to, they know who their GP is.'* (CH_Nurse_02)

They described how they had observed the interactions between patient and GP, which gave reassurance to the older adult, who had not only the GP but also their carer with them at the whole consultation during the lockdown.

There was a perception from care home staff that a large proportion of the residents preferred not to visit the surgery even before the pandemic. This was mainly owing to the difficulties associated with organising transportation to and from the home, and the associated organising and waiting periods for the transport. It was obvious to the care home staff that the journey to the surgery was stressful for the older adult patients. Therefore, for older adults who were able to comprehend the technology, using AVC was more convenient:

*'A few of them just find it stressful, you know, they’re waiting for transport to get there and then waiting for transport coming back. Sometimes they are waiting for transport for over half an hour. I think the waiting, they get a bit agitated over. I think from the residents, they don’t really like to go out to the surgery, they are happier for that sort of thing. There is a bit of workload trying to set it up the device, but it’s a lot better, I think with time you get used to it like anything else.'* (CH_Carer_02)

All participants seemed to agree that the quality of care using AVC was equivalent to face-to-face consultations. This is because the GP was able to confirm the vital observations of the patients in real time, which aided diagnosis. GPs described several advantages of real-time examination, including that the AVC facilitated an *'equally as good if not better'* mechanism for diagnosis and clinical decision-making for the GPs:

*'I think not only are they getting a more timely assessment, they’re also getting a very good quality assessment that’s equal to a lot of the things we would have actually routinely physically visited the care homes before. Er, and I don’t actually think that the actual clinical outcome would have been any different, so I think it’s equally as good if not better …'* (CH_DR_03)

Care home staff specifically indicated that illnesses such as chest infections and skin abnormalities were being diagnosed and treated earlier owing to the use of the AVC.

### Person-centred care in meeting the needs of older adults in care homes

The primary objective of a GP at any time is to ensure that a patient receives the treatment they need, but during a pandemic another key consideration is reducing secondary care referrals where possible. One GP mentioned that COVID-19 might potentially make residents less able to access medical care, as most routine visits had to be suspended. Some participants felt that the use of AVC and the tools provided to carry out remote observations meant that the residents received an enhanced quality of care from their GP as they were being seen, diagnosed, and treated much faster. In addition, the care home staff felt more attention was being paid to the resident's medical history:

*'I think it makes the doctor look at the medical history as well. Where, you know, before they would just diagnose over the phone and say, "Oh, just monitor them for a few days." Whereas now, because they are having to log in and look at them, I feel like they are looking more at their history as well. You know, the GPs are looking for different things probably to what we’re looking for sometimes when we report a patient condition to them.*' (CH_Carer_03)

### Optimising remote consultation and physical examination of older adults in care homes

The GPs and care home staff identified several factors that they believed could optimise the use of remote consultation and enable an effective AVC for older adults in care homes. Typical technological issues, such as poor Wi-Fi reception and poor connectivity, undermined the use of the technology in the care homes. As a result, not all care homes have been able to take full advantage of the remote consultation technology, and some residents missed out on the benefits of AVC, which could have undermined the trust in the technology. Another consideration is the appropriateness of remote consultation for residents with dementia and those receiving end-of-life care:

*'… This afternoon I’ve just been to a home to see someone* [in person] *who’s not got many hours to live. Just because, actually it’s nicer to actually see them with their relatives than do it over an AVC when you’re doing very difficult consultations.'* (CH_DR_02)

Some participants described how older adults with cognitive impairment would get disoriented when communicating with GPs via AVC. As a result, this often created challenges for staff who had to explain what was happening to the resident:

*'When you have technology involved with people with dementia, they don’t always know who is talking to them …'* (CH_Nurse_06).

Despite this, the GP would still be able to carry out the observation remotely via camera.

In general, the AVC has been well received. There were no reports of substantial disadvantages, except for technological issues such as internet reception. Specifically, the possibility to conduct real-time remote physical examinations was seen as a benefit. However, some of the GPs reported that it would be beneficial if the AVC software allowed storage of patient observation data. In this way, the GP could review the observations that were taken by the care home staff previously and confirm if there is a trend in the data:

*'At the moment the software doesn’t allow for the observations to be stored in the device so you can examine the trends. It really needs to have that capability for the carers to use it more proactively.* […]*'* (CH_DR_03).

One GP noted that some carers had trouble grasping the technology. As a result, this limited the use of the observational tools, as carers did not know how to operate them. In addition to not all care homes being able to use AVC regularly because of connectivity issues, another GP mentioned that not all staff felt comfortable using it because they were not acquainted with it or trained to use Medicspot. One GP even went out to provide some extra training themselves:

*' … we phoned a home the other week and the person who was there had forgotten how to use it* […] *so we all need reminding how to use it. And my colleagues do as well. So, the main thing is user familiarity and having connection.'* (CH_DR_02)

*'So recently I’ve actually driven out to all of our care homes and essentially done a test call with each of the partners who are looking after the care homes. So, I’ve been at the care home with the terminal, going around with the carers and actually retraining them and retraining myself on the job about how to use the terminal.'* (CH_DR_05)

Nevertheless, all participants reported that they did see the usefulness of the technology and would continue its usage post-lockdown.

## Discussion

### Summary

AVC showed great potential to improve accessibility of primary care services for older adults in care homes during a pandemic. GPs and care staff identified several advantages of using AVC, which was preferred to telephone consultations or, in some cases, in-person visits to the GP surgery. The specific AVC device allowed for some earlier diagnoses and treatment plans.

### Strengths and limitations

The strength of this study is in its exploration of the experiences of multiple stakeholders — GPs, care home nurses, and healthcare assistants — using AVC to provide healthcare delivery to older adults in care homes during the COVID-19 lockdown. Although AVC had several advantages, there were also challenges, including the appropriateness of remote consultation for residents with dementia and those receiving end-of-life care. Another key strength of the study is that the data collection took place while the city was in lockdown, thus providing highly relevant observations and minimising recall bias. A key limitation was the sample size of the GPs; however, in the UK the ratio of GPs to patients is approximately 0.46 to 1000, and in inner cities, the figures are even higher. Therefore, the number of GPs managing the care homes and who could participate in the study was adequate.

### Comparison with existing literature

The GPs and care home staff who participated in the study were positive regarding the use of virtual physical examinations of older adults in care homes during lockdown, as this aided the remote diagnoses and treatment for these older adults and facilitated better communication between the carers, GPs, and older adults, as reported in several other studies.^18,19,27^ In a previous study on video consultation for midwives, the midwives reported that video consultations demanded deeper engagement and presence compared with telephone consultations.^28^ According to the present study, the GPs and care home staff reported that the video consultations (more specifically, the physical examinations) promoted engagement both from the care providers and from the older adults when compared with telephone consultations, which had more typically been conducted between care home staff and GPs rather than patients and GPs.

The care home staff and GPs reported the benefits of virtual physical examinations in terms of facilitating more engagement of older adults during lockdown. This finding was also noted in a previous study,^20^ which also reported that AVC allows for more time and attention for patients with complex needs. Given the ongoing infection risk of SARS-CoV-2 to older adults, the use of AVC enabled timely health care for older adults during lockdown, which minimised their deterioration. This was important as several studies and National Institute for Health and Care Excellence (NICE) guidelines recommend timely prescriptions to offset deterioration, which would increase infection susceptibility.^9,29,30^

Another study on video consultations reported that patients who preferred video consultations noted the benefit of being in their usual familiar surroundings, while the presence of carers was deemed to assist with difficulties specific to the resident, such as hearing difficulties or loss of focus.^18^ Such findings are similar to those of the present study, whereby GPs and care home staff, where possible, included the older adults in the consultation. Similarly, among the benefits reported for video consultations is the decreased travel burden^17,18^ from the residence to the GP clinic and back, which is similar to the findings in this study. A key limitation of the AVC was the connectivity issues reported by care home staff and GPs — observations which have also been shared with the product developers.

### Implications for research and practice

The use of virtual physical examination could be an option for GPs and care home providers to aid prompt diagnoses, early intervention, and treatment for older adults who need medical attention, reducing the burden and stress of the older adults travelling to GP surgeries.

The use of AVC has the potential to allow GPs to create 'virtual ward rounds' and to reduce the risk of transmitting infectious illnesses, including COVID-19, winter flu viruses, and meticillin-resistant *Staphylococcus aureus* (MRSA), among older adults with underlying health conditions in care homes.

It is important that healthcare staff are involved in the development of the technology and that training is available to encourage confidence and competency in the implementation of the technology.

Further research is required to examine whether the use of remote physical examination could decrease GP visits to care homes without increasing the workload in GP services.

## Data

The dataset relied on in this article is available from the corresponding author on reasonable request.
